# Post-mortem oxycodone blood concentrations of hospitalized cancer and surgery patients compared with fatal poisonings

**DOI:** 10.1007/s00414-022-02890-2

**Published:** 2022-09-06

**Authors:** Pirkko Kriikku, Eija Kalso, Ilkka Ojanperä

**Affiliations:** 1 Forensic Toxicology Unit, Finnish Institute for Health and Welfare (THL), Helsinki, Finland; 2 Department of Forensic Medicine, University of Helsinki, Helsinki, Finland; 3 Department of Anaesthesiology, Intensive Care and Pain Medicine, Helsinki University Hospital, Helsinki, Finland; 4 Department of Pharmacology and SleepWell Research Programme, University of Helsinki, Helsinki, Finland

**Keywords:** Oxycodone concentration, Cause of death, Post-mortem toxicology, Treatment of cancer pain, Palliative care, Fatal oxycodone poisoning

## Abstract

Oxycodone is a strong opioid drug commonly used to treat acute, cancer, and chronic non-malignant pain. In this study, all oxycodone-related medico-legal cases where death had occurred in a hospital or nursing home in Finland were investigated to determine the range of post-mortem (PM) oxycodone blood concentrations in a therapeutic setting. All toxicology cases in which oxycodone was detected in PM femoral blood during the 4-year period of 2016–2019 in Finland were retrieved from the national PM toxicology database. In this material, the 365 deceased hospital patient cases that met the study inclusion criteria were divided into four groups according to the cause and manner of death. The reference group of 121 fatal oxycodone poisoning cases comprised two groups: those with verified associated drug abuse and those without drug abuse. The median oxycodone concentration in PM blood was significantly higher in cancer patients (0.10 mg/L) than in patients with recent surgery (0.07 mg/L) or other disease (0.06 mg/L) (*p* < 0.05). In addition, the median oxycodone concentration was significantly lower in all hospital patient groups than in the poisoning groups, the latter displaying 0.38 mg/L (abuse) and 0.64 mg/L (no abuse) (*p* < 0.001). This study shows that half of the subjects in the cancer patient group had PM blood oxycodone concentrations above the typical clinical therapeutic plasma concentration range (0.005–0.10 mg/L). Appropriate medication of hospitalized surgery and cancer patients can result in concentrations of up to 0.2 and 0.6 mg/L, respectively, while higher concentrations are exceptional.

## Introduction

Oxycodone is a semi-synthetic strong opioid analgesic commonly used to treat acute, cancer, and chronic non-malignant pain. The approximate equianalgesic morphine-to-oxycodone dose ratio is 1.5:1 following systemic administration. Compared with morphine, oxycodone has a faster onset of action, a better oral bioavailability, and a longer duration of action, but as a substrate of cytochrome P450 (CYP) enzymes, it is more prone to drug interactions than morphine. The most serious adverse effect of opioids in acute pain relief is respiratory depression. The dose should be appropriately balanced against pain, nausea, constipation, urinary retention, sedation, and itching [1]. Oxycodone is available as oral instant release and controlled release formulations as well as solutions for injection or infusion, either intravenously or subcutaneously.

CYP3A-mediated *N*-demethylation is the main metabolic pathway of oxycodone in humans. Substances that inhibit CYP3A4 may increase plasma concentrations of oxycodone, whereas those that induce CYP3A4 may decrease its plasma concentrations. The urinary metabolites derived from *N*-demethylation, namely noroxycodone, noroxymorphone, and α- and β-noroxycodol, account for 45% of the dose. The products from CYP2D6-mediated *O*-demethylation, namely oxymorphone and α- and β-oxymorphol, and from 6-keto-reduction, namely α- and β-oxycodol, account for 11% and 8% of the dose, respectively. However, the metabolites do not appear to contribute to central nervous system effects, either because of their low potency or low abundance in circulation or as a result of their poor uptake into the brain [2]. Particularly, oxymorphone is a potent opioid agonist and is marketed as a pharmaceutical product in the USA, but its plasma concentrations are very low after oxycodone administration. Noroxymorphone is also a potent opioid agonist but does not significantly cross the blood–brain barrier. The typical therapeutic plasma concentration range of oxycodone given in a standard reference work is 0.005–0.10 mg/L [3].

No opioid analgesic is without risk of misuse, abuse, and addiction, but oxycodone appears to have an exceptionally high likability and susceptibility to abuse [4]. In a medico-legal context, oxycodone is a relevant drug for many reasons. The US opioid epidemic, particularly the major role of oxycodone in opioid addiction and poisonings, has received much attention [5]. Oxycodone abuse and fatal accidental poisonings are to some degree a problem worldwide, and the drug also plays a role in suicides and undetermined deaths [6].

Less information is available about possible iatrogenic opioid poisonings in hospital inpatients [7]. Treatment-related deaths are often classified as natural, and they do not fall within the scope of medico-legal investigation and related post-mortem (PM) toxicology unless treatment is questioned by relatives or any other party. However, if an investigation into the cause of death is carried out, the focus is then on the alleged excessive amount of opioids given to the patient in the facility, possibly contributing to the death. The answer to this question is frequently sought by referring to the drug levels measured in PM blood taken at autopsy, but the lack of matched reference concentrations makes it difficult to interpret the results obtained [8].

In some jurisdictions, such as in Finland, the medico-legal autopsy rate is high and allows PM toxicology to be carried out in a broad range of indications, including appropriateness of pharmacotherapy. In the present study, our objective was to establish a distribution of oxycodone concentrations in PM blood that can be considered as normal after treatment of cancer, other disease, or recent surgery. We examined all oxycodone-positive Finnish medico-legal cases during the 4-year period 2016–2019 in which the individual had been treated in a hospital or a nursing home at the time of death and in which the manner of death was disease, occupational disease, or medicinal treatment or procedure. For comparison, we examined fatal oxycodone poisonings with or without history of drug abuse.

## Materials and methods

For this retrospective, register-based study, data were extracted from the forensic toxicology database maintained by the Finnish Institute for Health and Welfare (THL). The database contains all results from PM toxicological analyses in medico-legal investigations in Finland as well as information from death certificates. The study material consisted of the forensic pathologist’s referral, toxicology laboratory results, and information extracted from the death certificate. The death certificate, issued by the forensic pathologist who performed the autopsy, includes the cause and manner of death, substances implicated in fatal poisoning, with the primary finding separately indicated, a short description of the circumstances of death, and the autopsy findings.

Oxycodone was quantitatively determined in PM femoral venous blood from individuals who had died between 1 January 2016 and 31 December 2019. A validated and quality-controlled gas chromatography-mass spectrometry (GC–MS) method, operated in the selected ion monitoring mode with use of a deuterated internal standard, was employed for the analysis of oxycodone as part of a comprehensive toxicology panel. The limit of quantification for oxycodone was 0.02 mg/L. A general description of the toxicological panel used has been presented previously [9].

We included in the study those cases in which the patient had been treated in a hospital or a nursing home at the time of death and in which the manner of death was a disease, an occupational disease, or a medicinal treatment or procedure. Deaths during ambulance transportation were excluded. The cases meeting the inclusion criteria were divided into four groups: group 1: manner of death disease; cancer or malign tumors indicated. Group 2: manner of death disease; surgery associated with death. Group 3: manner of death medical treatment or procedure. Group 4: manner of death disease; no cancer or surgery. For comparison, all fatal oxycodone poisonings regardless of the manner and place of death over the same time period were included as reference cases and divided into two groups: group 5: fatal oxycodone poisonings related to drug abuse and group 6: fatal oxycodone poisonings not related to drug abuse.

All statistical analyses were carried out using IBM SPSS software (version 27). As frequency distributions of oxycodone blood concentrations and age of subjects were not normally distributed, medians and quartiles were used to characterize the data. The analyses were performed using the Mann–Whitney *U* test for independent samples. Comparisons between groups were done using Kruskal–Wallis *H* test, followed by the Dunn-Bonferroni test if a significant difference between groups was identified. A *p*-value < 0.05 was regarded as statistically significant.

## Results

All deaths between 2016 and 2019 in which the concentration of oxycodone in PM blood was above the limit of quantification were examined. Of these, we included in the study cases in which the patient had been treated in a hospital or a nursing home at the time of death and in which the manner of death was a disease, an occupational disease, or a medicinal treatment or procedure. The 365 cases meeting the inclusion criteria were divided into four groups: group 1: manner of death disease; cancer or malign tumors indicated (*N* = 70). Group 2: manner of death disease; surgery associated with death (*N* = 42). Group 3: manner of death medical treatment or procedure (*N* = 41). Group 4: manner of death disease; no cancer or surgery (*N* = 212).

In addition, all fatal oxycodone poisonings regardless of the manner and place of death over the same time period were included as reference cases and divided into two groups: group 5 — fatal oxycodone poisonings related to drug abuse (*N* = 64); group 6 — fatal oxycodone poisonings not related to drug abuse (*N* = 57).

**Fig. 1:**
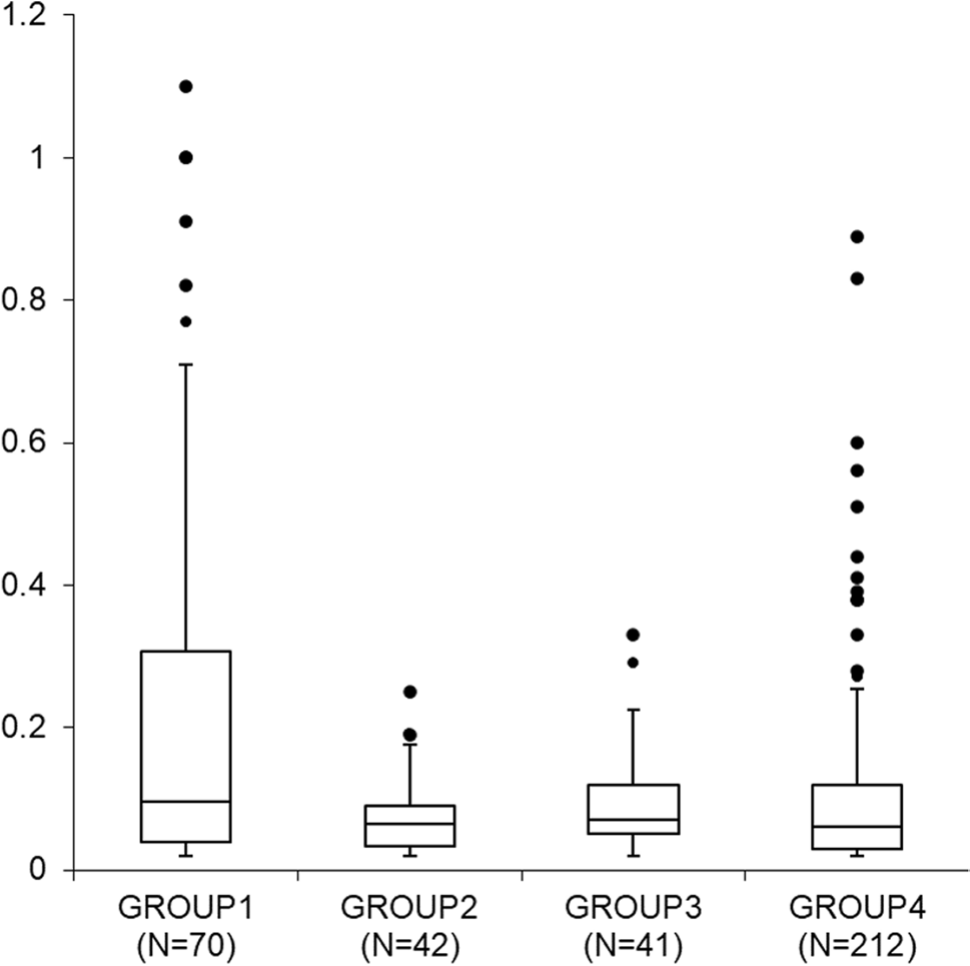
Concentration distribution of oxycodone in PM blood in hospital patient groups (groups 1–4). Boxes depict interquartile ranges, bars inside boxes median values, and whiskers the highest and lowest non-extreme values. Outliers (values higher than Q3 + 1.5*interquartile range) are given as dots above the boxes, except for the following high values: group 1: 2.2 mg/L, group 3: 1.3 mg/L, and group 4: 5.5 mg/L. Group 1: manner of death disease; cancer or malign tumors indicated (*N* = 70). Group 2: manner of death disease; surgery associated with death (*N* = 42). Group 3: manner of death medical treatment or procedure (*N* = 41). Group 4: manner of death disease; no cancer or surgery (*N* = 212)

Demographic information on the subjects and the summary distribution of oxycodone PM blood concentrations in the respective groups are given in Table 1. Differences between the oxycodone PM blood concentrations in the hospital patient groups (groups 1–4) are illustrated in Fig. 1.

**Table 1:** Demographic characteristics and oxycodone post-mortem (PM) blood concentrations in hospital patient groups (groups 1–4) and oxycodone poisoning groups (groups 5 and 6)^a^

	Group 1	Group 2	Group 3	Group 4	Group 5	Group 6
*N*	70	42	41	212	64	57
Median age (years)	71.0	78.5	70.5	77.5	33.0	57.0
Males (%)	61	62	46	50	80	44
Concentration, median (mg/L)	0.10	0.07	0.07	0.06	0.38	0.64
Concentration, 10th %tile (mg/L)	0.03	0.03	0.02	0.02	0.08	0.14
Concentration, 90th %tile (mg/L)	0.63	0.17	0.16	0.21	1.4	3.0
Concentration, range (mg/L)	0.02–2.2	0.02–0.25	0.02–1.3	0.02–5.5	0.02–47	0.04–48

^a^Group 1: manner of death disease; cancer or malign tumors indicated (*N* = 70). Group 2: manner of death disease; surgery associated with death (*N* = 42). Group 3: manner of death medical treatment or procedure (*N* = 41). Group 4: manner of death disease; no cancer or surgery (*N* = 212). Group 5: fatal oxycodone poisonings related to drug abuse (*N* = 64). Group 6: fatal oxycodone poisonings not related to drug abuse (*N* = 57)

Most of the subjects in group 1 (84%) had cancer as the underlying cause of death. In the remaining cases, cancer was a contributing cause of death, while the underlying cause of death was some other disease, e.g., coronary artery disease. Also in group 2, cancer was indicated in 26% of cases, but a recent surgery separated them from group 1.

In group 3, a medical treatment or procedure deemed to have caused the death was one of the following: death induced by complications during or after surgery (37%); death induced by complications related to other treatment procedures (27%); and death related to adverse effects of medicinal treatment (37%).

Group 4 consisted of other disease-related deaths. In most of these cases, the underlying cause of death was a disease of the circulatory system.

The subjects in groups 5 and 6 died of fatal poisoning by oxycodone alone or together with other substances. In group 5, fatal poisoning was considered to be the result of drug abuse, judging from drugs-of-abuse findings in PM toxicology and/or background information confirming recent drug abuse. The manner of death in abuse-related oxycodone poisonings was accident (unintentional) in 77%, undetermined in 17%, and suicide in 6.3% of cases. In group 6, fatal oxycodone poisoning was not related to drug abuse. Of these cases, 58% were suicidal poisonings, 32% unintentional, and 11% undetermined.

Of the subjects in groups 5 and 6, eight (7%) were hospitalized at the time of death from oxycodone poisoning, and seven of them had recently been taken to hospital for the treatment of poisoning. The remaining one patient with a history of drug abuse was recovering from surgery but had taken own drugs in addition to those received from the hospital and eventually died of fatal oxycodone poisoning. The other individuals in these groups had been found dead mostly at home.

Among the hospital patient groups (groups 1–4), the median oxycodone concentration in PM blood was significantly higher in group 1 than in group 2 or group 4 (*p* < 0.05). The 90th percentile concentration was markedly higher in group 1 than in the other three groups (Table 1). In both fatal oxycodone poisoning groups (groups 5 and 6), the median oxycodone concentration was significantly higher than in any of the hospital patient groups (*p* < 0.001).

The proportions of males ranged between 46 and 62% in groups 1–4, but the differences were not significant (*p* > 0.05). The proportion of males was higher in group 5 and lower in group 6 than in any of the hospital patient groups. The proportion of males in group 5 was almost twice as high as in group 6 (*p* = 0.001).

The subjects in groups 1 and 3 were markedly younger than in the other hospital patient groups, but only the difference between groups 2 and 3 was statistically significant (*p* < 0.05). The subjects in groups 5 and 6 were significantly younger than in any of the hospital patient groups (*p* < 0.001).

## Discussion

The main purpose of our study was to investigate the distribution of oxycodone levels measured in PM blood in patients who died in hospital or nursing home, with a focus on cancer and surgery patients. The reference concentration data obtained are essential evidence when assessing whether a patient’s medication administered by health care professionals has been appropriate. While approximately 85% of cancer patients achieve adequate pain relief with opioids [1], there is also a risk of under-treatment and over-treatment, the latter of which occasionally emerges as a suspicion of opioid overdose and may result in medico-legal cause-of-death determination.

Our study shows that the oxycodone levels of group 1–4 patients, with median concentrations ≤ 0.1 mg/L in PM blood, fall within the range found in living subjects, as demonstrated with the following published clinical data for cancer pain and breast cancer surgery patients.

In a multicenter cross-sectional study, Andreassen et al. [10] investigated the pharmacokinetics of oxycodone in hospitalized adult cancer pain patients (*N* = 439), who received a median (range) total oxycodone dose of 80 (10–960) mg/day. They reported median (range) serum oxycodone concentrations of 0.031 (< 0.0001–0.60) mg/L and concluded that women had lower oxycodone serum concentrations than men and that CYP3A4 enzyme inducers and inhibitors should have a significant impact on oxycodone pharmacokinetics. Another study by the same research group [11] examined the relationship between oxycodone and metabolite serum concentrations and the clinical effects in adult cancer pain patients. After an oral median (range) total oxycodone dose of 75 (10–1600) mg/day (*N* = 439), the median (range) serum oxycodone concentration was 0.033 (0.00032–0.60) mg/L; after a subcutaneous median (range) total oxycodone dose of 60 (15–390) mg/day (*N* = 12), the median (range) serum oxycodone concentration was 0.072 (0.031–0.25) mg/L; and after an intravenous median (range) total oxycodone dose of 348 (10–840) mg/day (*N* = 5), the median (range) serum oxycodone concentration was 1.1 (0.14–1.1) mg/L. The authors concluded that no relationships between oxycodone or noroxymorphone serum concentrations and pain intensity, tiredness, nausea, or cognitive function were present in this cross-sectional cohort of cancer patients.

Cajanus et al. [12] investigated factors affecting analgesic oxycodone concentrations in the immediate postoperative phase of breast cancer surgery. Oxycodone plasma concentrations at the first state of satisfactory analgesia varied greatly between individuals. In the moderate-motion pain intensity score group, after an intravenous mean (range) total oxycodone dose of 0.10 (0.02–0.6) mg/kg (*N* = 699), the mean (range) plasma oxycodone concentration was 0.032 (< 0.0001–0.31) mg/L. In the high-motion pain intensity score group, after an intravenous mean (range) total oxycodone dose of 0.18 (0.03–0.6) mg/kg (*N* = 170), the mean (range) plasma oxycodone concentration was 0.051 (0.0096–0.13) mg/L. CYP2D6 and CYP3A genotypes did not affect analgesic concentration or duration of analgesia.

We included fatal oxycodone poisonings as separate groups in our study because we wanted to see whether it was possible to set a limit between therapeutic and toxic PM concentrations. In the fatal oxycodone poisoning groups, consisting of drug abusers’ poisonings (group 5) and other oxycodone poisonings (group 6), the median oxycodone concentration was 0.38 and 0.64 mg/L, respectively, being significantly higher than in any of the hospital patient groups. In group 6, the proportion of suicides was high (58%), which may explain the even higher concentrations than in group 5. However, as shown in Table 1, the therapeutic and fatal PM concentration ranges overlap. The above-mentioned clinical studies indicate that opioid doses and the corresponding plasma concentrations in tolerant patients can be much higher than average, in surgery patients up to 0.3 mg/L and in cancer patients up to 1.1 mg/L, overlapping with the PM poisoning groups. Based on this, it is impossible to set a general cut-off value between therapeutic and toxic concentrations; the background information of each case must be separately considered. However, it can be stated that concentrations above 0.2 and 0.6 mg/L are exceptionally high in hospitalized surgery patients and cancer patients, respectively.

It should also be noted that today most opioid poisonings involve poly-drug use. Cone et al. [13] were among the first to draw attention to the fact that, based on oxycodone blood levels associated with single- and multiple-drug-induced fatalities, oxycodone in combination with other centrally active drugs was more toxic than when oxycodone was the only drug involved. They concluded that in cases of multiple-drug fatalities, the cause of death should not be attributed to any single drug but rather to the unique combination of drugs and individual factors [13]. The interactions may be pharmacokinetic, such as CYP3A4 inhibition by e.g. ritonavir and voriconazole, or pharmacodynamic, such as concomitant use of benzodiazepines.

As in our study, Jakobsson et al. [14] found a significant difference in oxycodone concentrations in PM femoral blood between groups where oxycodone contributed to fatal poisoning and in other causes of death. In their material, the median oxycodone concentrations were 0.042, 0.34, and 0.55 mg/L in non-intoxications (*N* = 93), mixed-intoxications (*N* = 48), and mono-intoxications (*N* = 20), respectively. These authors suggested that oxycodone concentrations exceeding 0.2 g/kg are likely to have contributed to toxicity, but concentrations as high as 0.3 g/kg might be encountered without toxic effects in a tolerant subject. They also did not recommend the use of a set oxycodone concentration to distinguish fatal intoxications. The authors focused on the concentration and metabolite ratio differences between various poisoning groups, but they did not specifically look at hospital patients as a distinct group.

PM blood is hemolyzed whole blood from which plasma or serum cannot regularly be separated. The PM redistribution phenomenon may increase or decrease drug concentrations after death, making interpretation challenging. Blood from peripheral sites, such as the femoral venous blood used in this study, is less affected by PM changes than blood from central sites. Previous studies have shown that prominent PM redistribution is commonly encountered with drugs with a high or low volume of distribution at steady state, including those with a blood-to-plasma ratio far from unity [15]. A high ratio of cardiac blood concentration to peripheral blood concentration of a drug is also commonly considered as a marker of PM changes [16]. In the case of oxycodone, several factors suggest that no significant PM change is likely to occur. Oxycodone has a moderate volume of distribution (2–4 L/kg) [1] and a blood-to-plasma ratio close to one (1.3) [17]. In terms of the PM cardiac-to-femoral blood ratio, oxycodone showed no consistent trend for significant PM redistribution [18, 19]. Further evidence against marked PM redistribution come from Finnish and Swedish studies that report a median oxycodone concentration of 0.08 mg/L (*N* = 3114) [9] and 0.085 mg/L (*N* = 189) [14], respectively, in PM femoral blood from oxycodone-positive autopsy cases representing all causes of death. These concentration levels are of similar magnitude to those found in living patients’ samples, as shown above.

A limitation of our study is that our concentration data consist only of the parent drug concentrations. However, according to Lalovic et al. [2], the central opioid effects of oral oxycodone are governed by the parent drug, with a negligible contribution from its circulating oxidative and reductive metabolites. This finding has since been confirmed by other studies that also after intravenous and epidural administration show active oxycodone metabolites to have only a minor contribution to the analgesia of oxycodone [20]. However, other authors suggest that metabolite analysis as well as genotyping may be of further assistance in differentiating between acute and chronic intake of oxycodone in suspected fatal poisonings [14, 21]. In any case, the practice of cause-of-death determination is affected by the fact that few PM toxicology laboratories analyze oxycodone metabolites quantitatively on a routine basis, and consequently, case interpretation regularly relies on the parent drug concentrations. Another limitation of our study is that we did not have sufficiently detailed information on patients who died in hospital to elucidate, for example, the effect of dose or timing of drug administration on concentrations.

We recognize that the limit of quantification of the analytical method used for our study (0.02 mg/L) did not allow determination of all the lowest oxycodone blood concentrations. However, this fact presumably has a very small effect on the statistics.

The consumption of oxycodone in Finland seems to have remained at a moderate level, in contrast to some other countries, especially the USA, where excessive prescribing of oxycodone to pain patients has triggered an opioid crisis [4, 22]. Drug consumption data, including hospital consumption, obtained from the Finnish Medicines Agency (FIMEA), expressed as defined daily doses (DDD) per 1000 inhabitants per day (DDD/1000 inhabitants/day), showed no changes in oxycodone consumption over the study period. In 2016–2019, the total DDD of oxycodone-alone products and the combination product containing naloxone varied between 1.75 and 1.82 DDD/1000 inhabitants/day with no trend up- or downwards. The share of hospital use in oxycodone-alone products and the combination product ranged between 25–28% and 32–40%, respectively, both figures showing a decreasing trend over the years.

Concerns raised by relatives regarding the use of opioids in hospital may be due in part to fears associated with the US opioid crisis. According to Sullivan and Ballantyne [23], the claim of a right to pain relief made by pain professionals, courts, and patient advocacy groups probably contributed to increased opioid prescribing, overdose deaths, and addiction problems. These authors emphasize that it was a mistake to extend the liberalization of opioid therapy from the end-of-life care to chronic pain care because end-of-life care occurs in a temporally and spatially circumscribed situation dominated by medical concerns.

There are challenges in establishing potentially toxic opioid levels in relation to both living and deceased subjects, owing to various disease conditions, polypharmacy, tolerance, and possible PM changes. Consequently, forensic pathologists and toxicologists must be very cautious in interpreting PM opioid drug concentrations [8, 24]. This is especially true after palliative care where the dose titrated to effect can be very high, balancing between relief of overwhelming pain and adverse events.

Finally, with a view to the future, we encourage more active monitoring of opioid levels in hospital patients, especially as less invasive and rapid analytical methods are being developed [25]. As regards the determination of the cause of death, for an improved future investigation of oxycodone poisoning, it is important to note that blood levels do not indicate brain concentrations, but analysis of PM brain tissue has recently been shown to be a viable diagnostic approach [26].

## Conclusions

In this study, we have provided evidence-based information for use by forensic practitioners and pharmacologists, especially in situations where the medication given to a patient in hospital or the results of cause-of-death determination are questioned. This study shows that the oxycodone concentration ranges in the PM blood of deceased hospitalized cancer or surgery patients are significantly different from those in the fatal oxycodone poisoning groups, although the ranges overlap. The median and 90th percentile oxycodone concentrations were 0.06–0.1 and 0.16–0.63 mg/L, respectively, in the hospital patient groups, and 0.38–0.64 and 1.4–3.0 mg/L in the poisoning groups. The similar oxycodone concentration ranges in blood between living and deceased hospitalized cancer or surgery patients, together with other pharmacological factors, suggest that no significant PM redistribution occurs with oxycodone.

## Data Availability

Not applicable.
